# Dynamic Micro-Batch and Token-Budget Scheduling for IoT-Scale Pipeline-Parallel LLM Inference

**DOI:** 10.3390/s26041101

**Published:** 2026-02-08

**Authors:** Juncheol Ahn, Yubin Son, Daemin Kim, Sejin Park

**Affiliations:** System Software Laboratory, Department of Computer Engineering, Keimyung University, Daegu 42601, Republic of Korea; 5645377@stu.kmu.ac.kr (J.A.); 5819937@stu.kmu.ac.kr (Y.S.); akqjq9250@stu.kmu.ac.kr (D.K.)

**Keywords:** large language models, IoT, edge computing, cloud inference, pipeline parallelism, micro-batching, GPU scheduling

## Abstract

Large language models in IoT–edge–cloud settings face bursty, heterogeneous requests that make pipeline-parallel inference prone to micro-batch imbalance and communication stalls, causing GPU idle time and SLO violations. We propose a runtime-adaptive scheduler that jointly tunes token budgets and micro-batch counts to balance prefill/decode workloads and minimize pipeline bubbles under changing compute and network conditions. On a four-node pipeline-parallel cluster across Llama-2-13b and Qwen2.5-14b at 100/1000 Mbps, our method outperforms vLLM and SGLang, reducing GPU idle time by up to 55% and improving throughput by up to 1.61 × while improving TTFT/ITL SLO satisfaction. These results show that dynamic scheduling is essential for scalable, latency-stable LLM inference in IoT–edge–cloud environments.

## 1. Introduction

Large language models (LLMs) are increasingly embedded into IoT, edge, and cloud services to support real-time analytics, semantic understanding, and interactive decision-making. Because IoT and edge devices lack sufficient compute capacity, their inference workloads are typically offloaded to cloud clusters, where pipeline parallelism is used to efficiently serve large numbers of concurrent requests.

However, real-world IoT workloads exhibit two challenging properties: (1) burstiness, where arrival rates fluctuate unpredictably, and (2) heterogeneity, where prompt and generation lengths vary significantly across devices and tasks. These characteristics create highly imbalanced mixtures of prefill- and decode-heavy requests. When such workloads flow through a pipeline-parallel LLM, even small imbalances across micro-batches propagate into substantial pipeline bubbles, inflating GPU idle time and destabilizing TTFT and inter-token latency (ITL).

A central—but often misunderstood—component of modern inference systems such as vLLM is the *token-budget* parameter. Contrary to common intuition, the token budget is not a throughput-optimization lever; rather, it is a *latency control knob* governing the TTFT–ITL trade-off. A small budget improves ITL but harms TTFT, while a large budget improves TTFT but increases ITL and prefill–decode interference. Static token-budget configurations therefore struggle to meet SLOs under dynamic IoT traffic and frequently exacerbate pipeline imbalance.

From a throughput perspective, the optimal condition would balance total computation across all micro-batches, allowing pipeline stages to progress in lockstep with minimal idle time. Yet this scenario is unattainable in practice due to unpredictable workload variability and strict TTFT/ITL SLO requirements that constrain how batches can be reshaped.

These limitations motivate the need for a scheduling mechanism that is both *dynamic* and *workload-aware*. In this work, we introduce two runtime-adaptive techniques: (1) *Dynamic Token-Budget Estimation*, which balances prefill and decode workloads across micro-batches while preserving TTFT/ITL constraints, and (2) *Dynamic Micro-Batch Scheduling*, which selects the micro-batch count that minimizes expected pipeline stalls under empirical compute and communication models.

We implement the proposed framework on a four-node RTX 4070 cluster using pipeline-parallel Llama-2-13b-chat with vLLM and evaluate it under both synthetic offline workloads and realistic online Poisson-arrival workloads. Our approach reduces GPU idle time by up to **55%**, improves throughput by up to **1.61×**, and achieves consistently higher TTFT/ITL SLO satisfaction compared with the baseline. These results demonstrate that dynamic scheduling is crucial for scalable and latency-sensitive LLM inference in IoT–edge–cloud environments.

## 2. Related Work

A significant body of prior work has investigated task placement, resource management, and inference scheduling for IoT and LLM systems. In this section we summarize the most relevant directions and highlight the remaining gap that motivates our work.

### 2.1. IoT and Edge–Cloud Computing Architectures

The IoT ecosystem increasingly relies on multi-tier architectures in which sensing, preprocessing, and decision-making operations are distributed across devices, edge nodes, and cloud servers. Edge computing is widely adopted to reduce the communication overhead and latency associated with offloading all computation to the cloud. Hamdan et al. survey edge-computing architectures for IoT applications and emphasize the importance of balancing latency, bandwidth savings, and computational capacity across layers of the system [1]. Andriulo et al. review edge–cloud hybrid computing frameworks and highlight that IoT workloads often exhibit high variability, requiring adaptive mechanisms to maintain efficiency and responsiveness [2]. These studies focus primarily on where tasks are executed within the IoT–edge–cloud hierarchy. Our work, in contrast, focuses on how to optimize the internal scheduling of LLM inference once the computation is already offloaded to a cloud or edge cluster.

### 2.2. Scheduling and Offloading in IoT and MEC Systems

Many prior works investigate task scheduling, resource allocation, and latency mitigation for IoT applications at edge or fog computing sites. Lim proposes a latency-aware scheduling method using AI-based task partitioning to optimize end-to-end inferencing delay in small-scale fog environments [3]. Eang et al. present a joint offloading and resource-allocation strategy in mobile edge computing (MEC) to balance latency and cost under real-time IoT constraints [4]. Saeik et al. survey mathematical, AI-based, and control-theoretic approaches for IoT task offloading across edge and cloud infrastructures [5]. These studies focus on global, multi-layer scheduling (device → edge → cloud) to reduce the total response time and network overhead. However, none of them address the fundamental computational bottleneck introduced by large language model inference inside edge/cloud clusters themselves. As IoT applications increasingly adopt LLM-powered analytics, the ability to serve inference requests at high throughput becomes essential. Our work is complementary to IoT offloading research: instead of deciding *where* to run tasks, we propose a method to improve *how* those tasks are executed inside the LLM-serving pipeline.

### 2.3. LLM Inference Systems and Pipeline Parallelism

Large language model inference introduces unique computational challenges due to the separation between the prefill and decode phases and the variability in request lengths. Systems such as vLLM [6], Orca [7], Sarathi [8,9], DistServe [10], and Splitwise [11] aim to reduce inference overhead through optimized batching, KV-cache management, and phase separation. Disaggregation-based approaches such as TetriInfer [12] and HexGen [13,14] explore architectural redesigns that separate model components to improve throughput under heterogeneous or mixed workloads. Pipeline parallelism is commonly employed to scale inference across multiple GPUs. Prior works such as GPipe [15] focus on pipeline efficiency in the training setting, but similar principles apply to inference. More recent efforts—including H_2_O [16], StreamingLLM [17], and FlashAttention variants [18,19,20]— optimize kernel execution, memory access, or attention mechanisms yet do not address system-level pipeline scheduling under highly variable request patterns. Our work differs from these approaches by targeting pipeline-stage imbalance caused by heterogeneous prompt lengths and decode workloads, a phenomenon particularly exacerbated by bursty IoT request patterns.

### 2.4. Throughput-Oriented Scheduling for LLM Inference

Recently, researchers have begun exploring throughput-driven resource management for LLM serving. POD-Attention improves prefill–decode overlap [21], while ExeGPT introduces constraint-aware scheduling for multi-tenant inference [22]. DeepSpeed-FastGen and Sequoia propose enhancements for speculative decoding [23,24], but their focus remains on token-level efficiency rather than pipeline-level throughput stabilization. Our method complements these methods by addressing a different dimension: the alignment of micro-batches and token budgets across pipeline stages to minimize GPU idle time in a multi-GPU setting. While speculative decoding or kernel optimization improves per-token efficiency, our scheduler improves per-step pipeline utilization—a critical requirement when serving high-volume IoT workloads.

### 2.5. Summary and Research Gap

Across IoT system research and LLM inference optimization, a clear gap emerges. IoT and MEC studies optimize where computation should occur (device ↔ edge ↔ cloud). LLM inference research optimizes computation inside the model. However, none of these studies address how to maintain stable throughput under IoT-scale workload variability in a pipeline-parallel LLM-serving cluster. Our work fills this gap by introducing a dynamic token-budget and Micro-Batch Scheduling framework that improves internal pipeline utilization and consequently enhances SLO stability for IoT applications relying on cloud-hosted LLMs.

## 3. Background

Maximizing GPU utilization in LLM inference requires a precise understanding of how workloads flow through the pipeline and where pipeline bubbles arise. Modern inference engines such as vLLM [25] typically employ pipeline parallelism to split a large model into multiple stages across GPUs, as well as micro-batching to keep those stages busy. However, when the amount of work in each micro-batch is imbalanced, or when inter-GPU communication latency becomes non-negligible, some stages finish earlier than others and wait idly, degrading end-to-end throughput.

**Figure 1:**
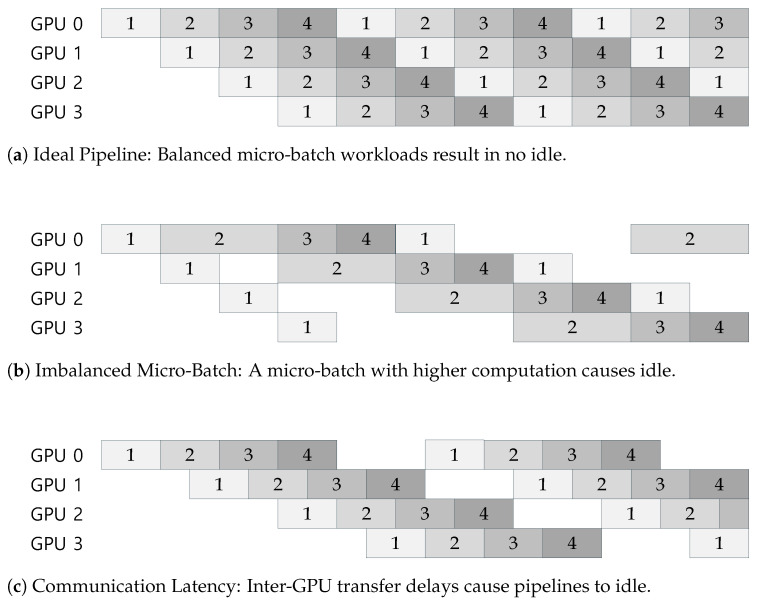
Pipeline execution scenarios with four micro-batches across four GPU ranks: (**a**) ideal, (**b**) with workload imbalance, and (**c**) with communication latency.

Figure 1 summarizes these effects with three representative scenarios:

- **(a) Ideal Pipeline:** Assuming uniform compute per micro-batch and ignoring inter-GPU communication latency entirely, the pipeline keeps every GPU fully utilized, reaching its theoretical peak throughput.
- **(b) Micro-Batch Imbalance:** In production workloads, *prefill* and *decode* token lengths vary between requests. A compute-heavy micro-batch creates a bottleneck that propagates down the pipeline, forcing later-stage GPUs to wait, and drastically reducing overall throughput.
- **(c) Communication Latency:** In multi-node deployments, network latency accumulates as micro-batches move between pipeline stages. Because LLM decoding is autoregressive, the first GPU cannot start the next micro-batch until the last GPU finishes the current one, aggravating idle time.

In the ideal case, micro-batches move like items on a conveyor belt, and each GPU stays busy during almost the entire pipeline cycle. In contrast, a single compute-heavy micro-batch or high communication latency can stall downstream stages and introduce long idle periods, even when total GPU compute capacity is sufficient.

These issues become more pronounced in emerging IoT/edge/cloud deployments. IoT devices and edge nodes generate requests with highly variable prompt lengths and bursty arrival patterns [1,2]. Inference is typically offloaded to a cloud or edge GPU cluster, where many devices share the same pipeline-parallel LLM backend. From the perspective of the pipeline, IoT traffic appears as a sequence of mixed-length prefill and decode workloads that arrive at irregular intervals. Consequently, it is difficult to maintain balanced micro-batches and fully utilized GPUs unless the scheduler explicitly accounts for these characteristics. The rest of this section reviews the key architectural features that interact with our proposed scheduling method: the asymmetry between prefill and decode, the behavior of the KV cache, and the role of the token-budget parameter.

### 3.1. Prefill and Decode Characteristics

LLM inference comprises two distinct computational phases:

- **Prefill:** The model processes the entire prompt and builds the key–value (KV) cache for all input tokens across all layers. This phase is highly parallel and compute-intensive because self-attention must consider all previous tokens in the prompt. As a result, its *computational* demand and memory footprint are substantial.
- **Decode:** By leveraging the cached KV tensors, the model generates one token at a time in an autoregressive manner. Each decode step only processes the newly generated token while attending to the existing KV cache. Thus, per-step computation and memory usage are significantly smaller than in the prefill phase, but must be repeated many times.

This structural asymmetry causes significant *variations* in per-request workload. Requests with long prompts but short outputs are dominated by prefill costs, whereas chat-like sessions with many generated tokens are dominated by decode processes. In pipeline-parallel execution, multiple requests and micro-batches are often processed concurrently on different pipeline stages. If the mixture of prefill- and decode-heavy requests is skewed across micro-batches, some stages finish much earlier than others and wait for the slowest micro-batch to complete, creating pipeline bubbles, as illustrated in Figure 1.

### 3.2. KV-Cache and Memory Preemption Issue

Transformer-based LLMs retain the KV cache in GPU memory to avoid recomputing attention for past tokens during decoding. While this design is essential for throughput, it also introduces complex memory-management challenges. The KV cache grows with both prompt length and the number of generated tokens. When many long-context or long-generation requests coexist, the aggregate KV-cache footprint can approach or exceed the available GPU memory capacity.

To cope with this pressure, inference engines rely on block-level KV-cache management policies, such as LRU or LFU-style eviction, and sometimes offload rarely used blocks to host memory or remote GPUs. However, these mechanisms are fundamentally reactive: once a block is evicted or migrated, some of the previously computed work is partially lost and must be recomputed if the corresponding request becomes active again. From a pipeline perspective, frequent KV-cache eviction or migration can propagate as additional stalls and bubbles, because downstream stages must wait for recomputation or data transfer to complete before proceeding.

In IoT and edge scenarios, where request burstiness and context lengths vary substantially across time and devices, KV-cache usage tends to fluctuate strongly. This makes it even more important to design scheduling strategies that avoid creating extreme imbalances in per-micro-batch KV-cache usage, since such imbalances amplify the likelihood of eviction and the associated recomputation overhead.

### 3.3. Token Budget: A Latency Control Parameter for Maintaining TTFT–ITL SLOs

Modern LLM inference systems such as vLLM [25] expose the *token-budget* parameter, which limits the maximum amount of computation allowed per micro-batch during decoding. Importantly, the token budget is **not a throughput-optimization mechanism**. Instead, it is a **latency-oriented control knob** designed to regulate the trade-off between the *Time To First Token* (TTFT) and *Inter-Token Latency* (ITL), enabling the system to satisfy application-level SLOs.

Operationally, the token-budget constrains how many tokens from all active requests can be processed in a single micro-batch. A smaller budget forces the scheduler to process fewer tokens per batch, whereas a larger budget allows more tokens to be advanced at once. This leads to the following fundamental latency behaviors:

- **Small token budget (ITL friendly and TTFT unfriendly):** With fewer tokens per micro-batch, each decode iteration is lightweight, reducing ITL. Furthermore, when a prefill request arrives, the scheduler can insert its prefill chunk between decode batches with minimal disruption, mitigating *prefill–decode interference*. However, because each micro-batch carries little work, the first token arrives later, increasing the TTFT.
- **Large token-budget (TTFT friendly, and ITL unfriendly):** Larger budgets enable more prefill tokens to be processed at once, improving the TTFT for long-prompt requests. However, inserting large prefill chunks into an ongoing decode stream causes significant prefill–decode interference, increasing ITL for existing decoding sessions and potentially amplifying queueing delays.

From a throughput perspective, the ideal scenario is to achieve *a perfectly balanced workload distribution* across all micro-batches. If the total computational load of prefill and decode were evenly partitioned across micro-batches, each pipeline stage would process similar amounts of work per cycle, eliminating pipeline imbalance and maximizing GPU utilization. However, this idealized scenario is unattainable in real systems for two reasons:

1. **Real-time workloads fluctuate unpredictably.** IoT and edge applications generate highly variable prompt lengths, decode lengths, and arrival patterns, causing sudden shifts in workload composition.
2. **The TTFT and ITL must remain within strict SLO bounds.** Allowing unrestricted batch growth destabilizes both the TTFT and ITL, especially under bursty or prefill-heavy workloads.

Thus, in existing systems, a *static* token budget is chosen as a compromise to balance the TTFT and ITL. However, static budgets fail to adapt to real-time changes in workload characteristics, making them suboptimal both for both SLO satisfaction and throughput stability.

**This limitation motivates our approach.** By dynamically estimating token budgets based on the current mixture of prefill and decode workloads, the proposed scheduler maintains TTFT/ITL SLOs **while also improving throughput**, reducing pipeline imbalance and minimizing GPU idle time. In other words, Dynamic Token-Budget Estimation transforms what was previously a static latency knob into an adaptive mechanism that simultaneously preserves SLOs and enhances effective system throughput under IoT-scale workloads.

## 4. Proposed Method

This section introduces two **runtime-adaptive scheduling techniques** designed to minimize **GPU Idle Time** in pipeline-parallel LLM inference. Our approach preserves the conventional pipeline execution model but enhances it with two adaptive mechanisms: (1) *Dynamic Token-Budget Estimation*, which balances compute load across micro-batches, and (2) *Dynamic Micro-Batch Scheduling*, which selects the optimal number of micro-batches to reduce communication-induced stalls. Together, these techniques maintain the TTFT/ITL trade-off while improving end-to-end throughput without modifying the underlying model or decode semantics.

**Design rationale and evidence.**

**Figure 2:**
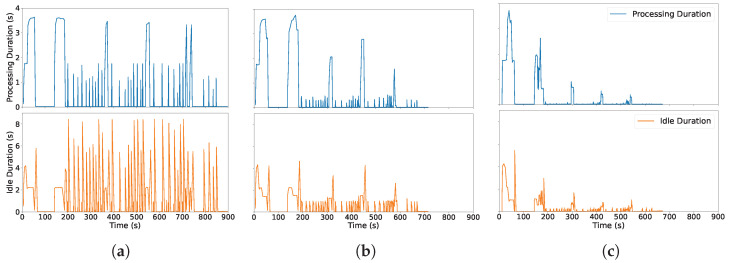
Processing and idle time timelines for the 64/1024/1024 scenario. (**a**) Baseline, (**b**) dynamic token budget, (**c**) dynamic token budget and micro-Batch.

**Figure 3:**
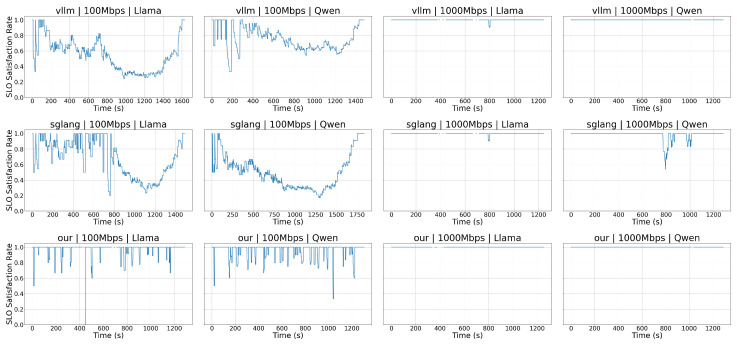
SLO satisfaction rates over time across systems, bandwidths, and models. Rows (top to bottom): vLLM, SGLang, and proposed method. Columns (left to right): 100 Mbps + Llama, 100 Mbps + Qwen, 1000 Mbps + Llama, and 1000 Mbps + Qwen.

As illustrated in Figure 1b,c, pipeline bubbles mainly stem from (i) *micro-batch compute imbalance* and (ii) *exposed communication stalls* when computation cannot hide inter-stage transfers. Accordingly, our design decomposes the problem into two coupled decisions, Dynamic Token-Budget Estimation targets (i) by equalizing per-micro-batch work, and Dynamic Micro-Batch Scheduling targets (ii) by selecting te micro-batch count that best matches the current computation–communication regime. Although the optimal policy is workload-dependent and combinatorial, our decision rules are derived from lightweight cost models and are validated by ablations and sensitivity results in Section 5 (Table 1, Figure 2 and Figure 3).

### 4.1. Dynamic Token-Budget Estimation

Schedulers that rely on a *fixed* **token budget** often distribute prefill and decode workloads unevenly, resulting in micro-batch imbalance. Under prefill-heavy workloads, this imbalance increases the likelihood of **preemption**, where an in-flight prefill is aborted and its KV cache is discarded, wasting already-computed work and exacerbating pipeline stalls.

To mitigate this, we *dynamically adjust the token budget per micro-batch based on the estimated compute cost*. Unlike static pre-chunking strategies, our method assigns at most *one* prefill chunk to each micro-batch and distributes the overall prefill workload evenly across num_micro_batch.

This choice is motivated by two key observations:

- Each prefill generates KV-cache tensors that occupy GPU memory. Scheduling many small prefills concurrently increases memory pressure and raises the risk of preemption. A dynamic token budget naturally limits concurrent the prefill inflight size, lowering this risk.
- Splitting a prefill into numerous small chunks can benefit decode-heavy workloads, but excessively small chunks inflate the TTFT and make GPU compute saturation difficult. By evenly dividing the overall prefill cost across exactly num_micro_batch, we maintain a consistent TTFT/ITL balance while improving pipeline utilization.

The effect on pipeline behavior is intuitive: if the first micro-batch requires *x* seconds and the next requires y>x, then a *k*-stage pipeline may incur up to (k−1)(y−x) idle time (Figure 1b). Reducing the compute gap between consecutive micro-batches—by equalizing their workloads—significantly decreases such stage-amplified idle propagation.

By introducing Dynamic Token-Budget Estimation, the TTFT can be reduced without sacrificing ITL, because the micro-batch count continues to regulate the TTFT/ITL trade-off. Algorithm 1 formalizes this estimation procedure, separating prefill and decode workloads and balancing them across micro-batches to minimize GPU idle time.
**Algorithm 1:** Dynamic Token-Budget Estimation
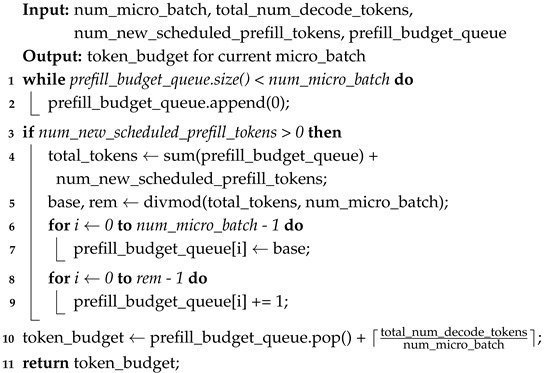

**Why this can outperform static token budgets.**

Let $T_{\mathrm{prefill}}$ be the total newly scheduled prefill tokens in a step and partition them into k=num_micro_batch parts $\left\{t_{1},\ldots ,t_{k}\right\}$. When prefill cost is non-decreasing and approximately proportional to token count, minimizing $\max_{j}t_{j}$ reduces the worst-case micro-batch runtime, which directly limits stage-amplified idle propagation (cf. Figure 1b). A near-uniform split $t_{j}\in \left\{\left\lfloor T_{\mathrm{prefill}}/k\right\rfloor ,\left\lceil T_{\mathrm{prefill}}/k\right\rceil \right\}$ achieves this objective, motivating Algorithm 1.

**Evidence in our setting.**

Static token budgets in vLLM may repeatedly chunk large prefills and trigger preemption, wasting already-computed KV-cache work. In the offline results (Table 1), Dynamic Token-Budget reduces idle time and redundant processing in long-sequence workloads (e.g., 64/1024/1024 and 64/2048/2048), indicating that balancing prefill load mitigates both bubble amplification and re-execution overhead.

### 4.2. Dynamic Micro-Batch Scheduling

Even with balanced micro-batches, pipeline efficiency still depends critically on the *number of micro-batches*. Given empirical models of per-batch computation and communication cost, the scheduler must choose the value of num_micro_batch that minimizes the expected number of pipeline stalls.

Assuming balanced workloads—achieved by Dynamic Token-Budget Estimation—the following two timing components characterize pipeline efficiency:

- **First-Token Generation Time**
  (1) $$T_{\mathrm{first}\text{\_}\mathrm{token}}=\mathrm{comp}\text{\_}\mathrm{time}\times \mathrm{pp}\text{\_}\mathrm{size}+\;\mathrm{comm}\text{\_}\mathrm{time}\times \left(\mathrm{pp}\text{\_}\mathrm{size}-1\right)$$
  representing how long it takes for the first micro-batch to traverse the entire pipeline.
- **Rank-0 GPU Total Time**
  (2) $$T_{\mathrm{rank}0}=\mathrm{comp}\text{\_}\mathrm{time}\times \mathrm{num}\text{\_}\mathrm{micro}\text{\_}\mathrm{batch}$$
  representing how long the first GPU remains busy processing all micro-batches.

The absolute difference

$$\left|T_{\mathrm{first}\text{\_}\mathrm{token}}-T_{\mathrm{rank}0}\right|$$

captures how much idle time is created on downstream pipeline stages. **Dynamic Micro-Batch Scheduling** selects the number of micro-batches that minimizes this gap. This procedure is shown in Algorithm 2.
**Algorithm 2:** Dynamic Micro-Batch Scheduling
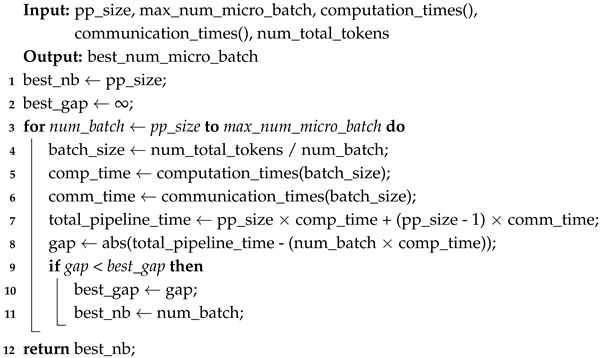

**Rationale: the gap as a proxy for exposed stalls.**

With balanced micro-batches, each stage iterates over (i) computation and (ii) inter-stage transfer. If computation per micro-batch is too small relative to communication, transfers cannot be hidden, creating bubbles on downstream stages. $T_{\mathrm{first}\text{\_}\mathrm{token}}$ captures how fast the first micro-batch reaches the tail stage, while $T_{\mathrm{rank}0}$ captures how long the head stage remains busy generating micro-batches. A large mismatch implies either premature idleness (head finishes too early) or insufficient overlap (downstream waits for data), both manifesting as idle time. Therefore, minimizing $|T_{\mathrm{first}\text{\_}\mathrm{token}}-T_{\mathrm{rank}0}|$ is a lightweight, model-based heuristic for reducing communication-induced stalls.

**Why this differs from prior dynamic chunking.**

SGLang focuses on correcting stage misalignment mainly induced by compute-side variability. Our method explicitly incorporates both computation and communication models and adapts num_micro_batch to the bandwidth regime, which is particularly beneficial in low-bandwidth deployments (validated in Figure 3).

When computation dominates or network latency is relatively small, the algorithm tends to select fewer micro-batches to avoid unnecessary fragmentation. When communication latency is significant, it increases the micro-batch count to improve overlap between computation and communication. This adaptive strategy sustains robust performance across varying workload conditions and pipeline depths.

### 4.3. Why Estimation and Scheduling Must Be Joint

The two components are intentionally co-designed. Dynamic Micro-Batch Scheduling assumes that micro-batches have comparable compute cost; otherwise, a micro-batch count selected from average comp_time(·) becomes unreliable due to imbalance-driven outliers. Dynamic Token-Budget Estimation, in turn, requires a micro-batch count *k* that matches the current communication regime: choosing *k* too large fragments work and inflates TTFT, while choosing *k* too small exposes communication stalls and increases idle time. Therefore, the effective solution is a closed-loop policy that (1) selects *k* using compute/communication profiles and (2) estimates balanced token budgets conditioned on *k*.

This coupling is also supported empirically: Table 1 shows that adding micro-batch adaptation on top of token-budget balancing consistently yields additional idle-time reduction, and Figure 2 visualizes how the joint design shrinks contiguous idle regions in long-sequence workloads.

## 5. Experiments

In this section, we evaluate the proposed **Dynamic Token-Budget** and **Dynamic Micro-Batch** Scheduling techniques on a real pipeline-parallel LLM inference system. We first describe the experimental environment and compared configurations, then present results for two scenarios: an *offline* setting with controlled synthetic workloads and an *online* setting that emulates real-time service conditions. Our analysis focuses on *GPU idle time*, *throughput* (completion time), and *latency SLO satisfaction*.

### 5.1. Experimental Setup

We evaluated the proposed techniques on a four-node **LAN cluster**. Each node is configured as follows: **CPU:** Intel Core i9-13900, **GPU:** NVIDIA RTX 4070 (12 GB), **Memory:** 64 GB RAM, **OS:** Ubuntu 22.04 LTS, **NVIDIA driver:** 570, and **CUDA:** 12.8, which uses a Docker-based runtime with Python 3.12.10. The inter-node network bandwidth is configured at **100 Mbps** and **1000 Mbps**. We use vLLM v0.9.0.1 orchestrated with Ray 2.46.0 and Transformers 4.52.4 (base image: vllm/vllm-openai:v0.9.0.1). The models under test are meta-llama/Llama-2-13b-chat-hf and Qwen2.5-14b, each partitioned into *four* pipeline stages—one stage per node. We compare **vLLM**, **SGLang** v0.5.7 (dynamic chunking enabled), and **our method** under the same workload generator.

Within vLLM, we report three configurations:

- **Baseline:** The stock implementation of vLLM v0.9.0.1 using its default static token-budget and Micro-Batch Scheduling policy.
- **Dynamic Token Budget:** Baseline augmented with *Dynamic Token-Budget Estimation* only.
- **Dynamic Micro-Batch:** The full version of the proposed method, combining *Dynamic Token-Budget Estimation* and *Dynamic Micro-Batch Scheduling*.

This setup reflects a realistic multi-node cloud/edge deployment where a pipeline-parallel LLM is served over networks ranging from constrained to high-bandwidth. The offline scenario reports the three vLLM-based configurations, while the online scenario reports all three systems (vLLM, SGLang, and our method).

### 5.2. Scenarios and Evaluation Metrics

We evaluate the three configurations in two complementary scenarios:

1. **Offline scenario:** Synthetic workloads with controlled request parameters are used to examine how the methods behave under different sequence lengths and concurrency levels.
2. **Online scenario:** Requests arrive according to a Poisson process, mimicking real-time service conditions with heterogeneous input/output lengths and time-varying load.

For each run we record three time-based metrics:

- **Completion time**: This is the total wall-clock time from the arrival of the first request until all requests are completed. This directly reflects throughput.
- **Processing time (Proc)**: This is the aggregated time during which GPUs are actively executing kernels.
- **Idle time**: This is the cumulative time during which at least one GPU has no work to execute (i.e., pipeline bubbles).

#### 5.2.1. Offline Scenario

In the offline scenario, we configure multiple workloads by varying the number of requests, input token lengths, and output token lengths. For each workload, we measure **completion**, **rocessing**, and **idle** times to compare the three scheduling methods. Table 1 summarizes the results, along with the improvement in the full **dynamic token-budget and micro-batch** scheme over the **baseline**.

Across *all* workloads, the two dynamic schedulers outperform the fixed baseline. For moderate request sizes such as 64/256/256, the full scheme reduces the **Completion** time from 130.3 s to 111.6 s (a **1.17× speed-up**) while cutting the **Idle** from 66.5 s to 47.7 s (–28%). As sequence length increases, the benefits become more pronounced: for 256/256/256, completion tine decreases from 616.2 s to 381.9 s (**1.61×**) and idle time drops by 55%. The longest prompt configuration 64/2048/2048 similarly improves from 2468.7 s to 2113.0 s (1.17×) while reducing idle time by 16%.

The main driver of improvement is reduced **GPU idle time**. In the 64/1024/1024 and 64/2048/2048 workloads, the baseline experiences repeated *chunking* and **preemption**, inflating idle times to 429.3 s and 415.7 s, respectively. The dynamic scheme limits preemption, reducing idle times to 353.4 s and 234.2 s. Longer sequences also reveal the impact on redundant computation: for 64/1024/1024, our scheduler cuts **Proc** from 475.6 s to 424.9 s (–11%), and for 64/2048/2048,it cuts Proc from 1684.4 s to 1451.1 s (–14%) by preventing repeated prefill re-execution.

Overall, the proposed schedulers reduce both compute imbalance and re-execution overhead, yielding up to **1.61×** faster completion times and as much as **55%** less GPU idle time without penalizing processing time.

To illustrate the effect more concretely, Figure 2 shows the timelines of GPU processing and idleness for the large-input workload 64/1024/1024. The baseline exhibits long, contiguous idle intervals, whereas the proposed methods progressively shrink idle regions and shorten the overall completion time.

#### 5.2.2. Online Scenario

To emulate a realistic service workload, we generate **128 requests** whose input and output lengths are drawn *uniformly at random* (input: 512–2048 tokens; output: 256–1024 tokens). Requests arrive according to a **Poisson process** with rate λ=0.1requests, and are dispatched according to order of arrival. This setting allows us to assess how well each scheduler preserves *latency quality*—i.e., the TTFT and ITL—under heterogeneous, time-varying loads.

Per-request performance is measured using an **SLO satisfaction rate** based on two latency metrics: the TTFT and ITL. Thresholds are set to reflect production responsiveness requirements; during each run we record the fraction of in-flight requests that satisfy both TTFT and ITL targets.

##### Sensitivity Analysis and SOTA Comparison

We run the online scenario under **100 Mbps** and **1000 Mbps** with two models (**Llama** and **Qwen**), and compare **vLLM**, **SGLang**, and **our method**. Figure 3 plots the SLO satisfaction ratio over time across all 12 combinations. Under **100 Mbps**, vLLM and SGLang exhibit frequent SLO violations, whereas our method maintains a consistently higher SLO ratio across both models. At **1000 Mbps**, all systems improve, and the performance gap narrows, indicating that the gain is strongest when communication bottlenecks dominate.

Overall, the proposed scheduler delivers higher SLO attainment under constrained bandwidths and remains competitive as bandwidth increases.

### 5.3. Results and Analysis

The experimental results confirm that the proposed **dynamic token-budget** and **dynamic micro-batch** techniques significantly enhance pipeline-parallel LLM inference. When combined, they reduce **GPU idle time** by up to **55%** and improve completion time (throughput) by up to **1.61×**. On average across all offline workloads, idle time drops by **36.2%** and throughput increases by **1.28×**.

These gains indicate that a runtime-aware model of computation and communication enables the scheduler to adapt to diverse token lengths and dynamic network delays. The improved SLO satisfaction observed in the online scenario further shows that our approach not only raises efficiency but also maintains real-time latency requirements, making it well-suited for latency-sensitive LLM services deployed in IoT–edge–cloud environments.

Finally, the two methods are *synergistic*: balancing compute load (**dynamic token-budget**) and adapting to communication constraints (**dynamic micro-batch**) together deliver higher performance than either technique alone.

## 6. Discussion

We now discuss the implications of our results for IoT-scale deployments and position our throughput-first design relative to latency-oriented approaches.

### 6.1. Effectiveness for IoT-Scale Workloads

IoT traffic is characterized by intermittent bursts and heterogeneous request sizes. Our results confirm that token-budget estimation effectively mitigates compute imbalances caused by diverse prompt lengths, while adaptive micro-batch scheduling smooths traffic variability. Pipeline stability is significantly improved: GPU idle time is reduced, and per-stage utilization remains high even as traffic patterns shift. These properties are essential for real-time IoT services that run LLM inference in the cloud, where sudden spikes in event-triggered requests can otherwise cause severe performance degradation.

### 6.2. Throughput-First vs. Latency-First Optimization

Traditional inference schedulers often target token-level latency metrics, such as minimizing TTFT or per-token generation delay for individual sessions. However, IoT backends typically need to sustain a large volume of concurrent data streams and maintain SLOs at the *system* level. In this context, throughput stability—rather than best-effort latency for a single request—is the primary requirement.

Our throughput-first design supports more devices per cluster, ensures more predictable latency under high loads, and improves system capacity without additional hardware. By explicitly maximizing pipeline utilization and reducing bubbles, the scheduler reduces queueing delay for many requests simultaneously. This perspective aligns naturally with IoT service requirements and distributed edge–cloud computing paradigms, where compute and network resources must be shared across large numbers of devices.

### 6.3. Comparison with Existing Dynamic Scheduling

Sarathi-Serve introduces chunked prefills to dynamically split requests and avoid long prefill stalls. This capability is already integrated into vLLM and is therefore reflected in our vLLM baseline. However, the chunked prefill still relies on a fixed token budget and does not adjust the micro-batch count to account for communication delay. SGLang’s dynamic chunking targets stage misalignment caused by non-uniform per-chunk runtime, reducing bubbles that arise from compute imbalance. Our approach complements these methods by explicitly modeling both computation and communication times and selecting the micro-batch count accordingly. In the low-bandwidth setting, this yields higher SLO satisfaction than SGLang while retaining the benefits of dynamic chunking.

### 6.4. Scheduler Overhead

The runtime overhead of our scheduler is minimal. Both algorithms use lightweight time estimates and a small search over micro-batch counts; in our implementation, the overhead is well below 1 ms per step, which is negligible relative to GPU kernel execution and inter-GPU communication.

### 6.5. Impact of Network Bandwidth

We evaluate both 100 Mbps and 1000 Mbps inter-node bandwidths to represent constrained and higher-bandwidth deployments.

At 100 Mbps, communication-induced stalls dominate, and the proposed scheduler substantially improves SLO satisfaction and throughput stability across both models. At 1000 Mbps, all systems improve, and the performance gap narrows, indicating that the largest gains appear when communication is the primary bottleneck.

Even in higher-bandwidth settings, the scheduler remains a safe choice because its overhead is negligible and it does not degrade performance.

Nevertheless, evaluating heterogeneous wide-area network settings remains an important direction for future work.

### 6.6. Generalization, Limitations, and Future Work

Both Algorithm 1 and 2 are lightweight and easily integrated into existing inference engines. Their designs are model-agnostic, making them applicable to a variety of LLM architectures, pipeline depths, and deployment scales, including future edge–cloud deployments and real production IoT workloads. The framework is not tied to vLLM and can be integrated into other pipeline-parallel LLM-serving systems with minimal changes.

Nevertheless, this study has limitations. Our experiments are conducted on a homogeneous GPU cluster with relatively stable intra-cluster network latency. Although this setting is representative of many cloud deployments, real-world edge–cloud systems often exhibit heterogeneous compute capabilities and non-negligible wide-area network delays. In such environments, the interaction between pipeline scheduling, network latency, and cross-layer offloading decisions becomes more complex. Despite this, the approach is compatible with heterogeneous sensing pipelines: by incorporating per-stage compute and communication profiles, the scheduler can coordinate pipeline-stage latencies and integrate with heterogeneity-aware model-partitioning strategies.

Despite these constraints, our results suggest that the proposed method already improves throughput in settings where network performance is a relative bottleneck, by shortening pipeline idle time and reducing unnecessary micro-batch stalls. A promising direction for future work is to extend the framework to heterogeneous clusters with large network delays and diverse GPU capabilities by jointly optimizing micro-batch scheduling, token budgets, and cross-node placement to maximize end-to-end throughput and SLO satisfaction.

## 7. Conclusions

As large language models are increasingly deployed in IoT–edge–cloud environments, maintaining both high throughput and stable latency becomes a central challenge. Bursty, heterogeneous workloads with mixed prefill- and decode-heavy requests easily amplify pipeline-stage imbalance in pipeline-parallel LLM inference, causing GPU idle time to grow and making it difficult to meet TTFT and ITL service-level objectives (SLOs) even when sufficient raw compute capacity is available. Static batching and token-budget configurations are ill-suited to such dynamic conditions, as they cannot react to real-time changes in workload composition or network behavior.

In this work, we presented a runtime-adaptive scheduling framework that combines *Dynamic Token-Budget Estimation* with *Dynamic Micro-batch Scheduling*. The proposed method uses token-budget estimation to balance prefill and decode workloads across micro-batches, and selects the number of micro-batches to minimize pipeline bubbles based on empirical compute and communication models. Crucially, we do not treat the token-budget as a fixed throughput knob; instead, we reinterpret it as a latency control parameter that can be adjusted at runtime to preserve TTFT/ITL SLOs while improving GPU utilization.

Our implementation on a four-node RTX 4070 cluster running pipeline-parallel Llama-2-13b-chat with vLLM demonstrates that the combined scheme reduces GPU idle time by up to **55%** and improves throughput (completion time) by up to **1.61×** compared with the baseline static scheduler. On average, across offline workloads, idle time is reduced substantially, and throughput increases by more than 1.2×. Furthermore, in an online Poisson-arrival scenario, the proposed framework maintains a higher TTFT/ITL SLO satisfaction ratio, especially during bursty periods and under network bottlenecks.

These results suggest that dynamic, workload-aware scheduling is a practical and effective way to unlock additional performance from existing LLM inference engines without modifying model architectures or kernels. Future work includes extending the framework to heterogeneous GPU clusters, incorporating richer network feedback, and jointly optimizing scheduling decisions with cross-layer offloading policies in IoT–edge–cloud systems.

## Figures and Tables

**Table 1:** Completion, processing (Proc), and GPU idle (Idle) times for each workload. The last column reports the gain of dynamic token budget + micro-batch over the baseline (speed-up for Completion; percentage reduction for Proc and Idle).

Workload (Req/In/Out)	Metric	Baseline	Only Dynamic Token-Budget	Dynamic Token Budget and Micro-Batch	Improvement
64/256/256	Completion	130.3 s	123.8 s	111.6 s	1.17×
	Proc	63.3 s	67.1 s	62.4 s	1%
	Idle	66.5 s	56.2 s	47.7 s	28%
128/256/256	Completion	290.9 s	226.4 s	211.0 s	1.38×
	Proc	112.6 s	110.8 s	122.4 s	−9%
	Idle	177.7 s	115.0 s	88.0 s	50%
256/256/256	Completion	616.2 s	441.0 s	381.9 s	1.61×
	Proc	206.3 s	197.2 s	197.9 s	4%
	Idle	409.3 s	243.2 s	182.6 s	55%
32/512/512	Completion	152.0 s	147.5 s	139.0 s	1.09×
	Proc	88.2 s	91.8 s	87.4 s	1%
	Idle	63.2 s	55.2 s	51.1 s	19%
64/512/512	Completion	319.3 s	268.7 s	256.5 s	1.24×
	Proc	158.8 s	158.4 s	170.7 s	−8%
	Idle	160.0 s	109.7 s	85.2 s	47%
128/512/512	Completion	673.5 s	499.9 s	471.7 s	1.43×
	Proc	295.0 s	266.0 s	282.8 s	4%
	Idle	377.9 s	233.4 s	188.3 s	50%
32/1024/1024	Completion	420.6 s	366.5 s	349.6 s	1.20×
	Proc	255.7 s	255.0 s	241.6 s	6%
	Idle	164.4 s	110.9 s	107.4 s	35%
64/1024/1024	Completion	891.8 s	708.8 s	660.5 s	1.35×
	Proc	475.6 s	447.5 s	424.9 s	11%
	Idle	415.7 s	260.7 s	234.2 s	44%
32/2048/2048	Completion	1262.9 s	1142.3 s	1092.5 s	1.16×
	Proc	833.1 s	782.9 s	738.6 s	11%
	Idle	429.3 s	358.8 s	353.4 s	18%
64/2048/2048	Completion	2468.7 s	2222.2 s	2113.0 s	1.17×
	Proc	1684.4 s	1567.0 s	1451.1 s	14%
	Idle	783.8 s	654.5 s	660.4 s	16%

## Data Availability

No new data were created in this study. Data sharing is not applicable to this article.
